# Health outcomes, education, healthcare delivery and quality – 3059. Relation between body mass index and control of bronchial asthma among Egyptian adults with asthma

**DOI:** 10.1186/1939-4551-6-S1-P226

**Published:** 2013-04-23

**Authors:** Mohamed Helmi Zidan, Rasha Daabis, Reem Ibrahim

**Affiliations:** 1 Department of Chest Diseases, Alexandria Faculty of Medicine, Alexandria, Egypt; 2 Department of Internal Medicine, Alexandria Faculty of Medicine, Alexandria, Egypt

## Background

A significant and concomitant increase in the prevalence of both asthma and obesity has occurred worldwide. Obesity has recently been identified as a major risk factor for the development of asthma. Asthma in the obese individual tends to be more severe, does not respond well to treatment and is becoming a major public health issue in many countries.Thus, recent literature suggests that Asthma in the obese may represent a unique phenotype of the disease that is often referred to as “obese-asthma” or “obesity-associated asthma”.

## Methods

Asthma control was determined using the asthma control questionnaire (ACQ) for 300 adult asthmatics patients. According to their BMI, patients were categorized into normal weight, overweight, obese and morbid obesity. Patients in the different BMI categories were compared for demographic and clinical characteristics as well as the ACQ scores. General linear models were used to evaluate the impact of BMI on asthma control (ACQ score).

## Results

64% of the asthmatic patients were either overweight or obese. Patients with higher BMI scores showed a statistically significant elevation in ACQ scores. This association between BMI and ACQ score persisted after adjusting for age, sex, duration of asthma, selected comorbidities and the use of asthma controller medications using general linear models. Also, patients with higher BMI reported more hospitalization, more oral steroid use and the use of more prescribed controller medications.

## Conclusions

Patients need not be classified as “obese” for excess weight and body fat to compromise several important asthma variables. Our findings suggest that higher BMI, irrespective of BMI “category”, may have important implications for asthma control, medication use, and asthma-specific hospitalization. Hence, suggesting important avenues for asthma management and control initiatives.

